# Supplementary material for: Reversible Redox Property of Co(III) in Amorphous Co-Doped SiO2/γ-Al2O3 Layered Composites
Source: Materials (Basel). 2020 Nov 25;13(23):5345. doi: 10.3390/ma13235345 (PMC7728299; doi:10.3390/ma13235345)
Supplement: Supplementary file 1 [file materials-13-05345-s001.pdf]

materials

Supplementary Materials: Reversible Redox Property of Co(III) in Amorphous Co-Doped $SiO_2/\gamma\text{-}Al_2O_3$ Layered Composites

Shotaro Tada [1], Shota Saito [1], Akito Mori [1], Hideki Mizuno [1], Shiori Ando [1], Toru Asaka [1], Yusuke Daiko [1], Sawao Honda [1], Samuel Bernard [2] and Yuji Iwamoto [1,*]

[1] Department of Life Science and Applied Chemistry, Graduate School of Engineering, Nagoya Institute of Technology, Gokiso-cho, Showa-ku, Nagoya 466-8555, Japan; s.tada.341@stn.nitech.ac.jp (S.T.); shota.saito@cgco.co.jp (S.S.); a_mori_10029@murata.com (A.M.); m.hideki0318@gmail.com (H.M.); shiori786@outlook.jp (S.A.); asaka.toru@nitech.ac.jp (T.A.); daiko.yusuke@nitech.ac.jp (Y.D.); honda@nitech.ac.jp (S.H.)

[2] Centre Européen de la Céramique, University of Limoges, 12 Rue Atlantis, 87068 Limoges, France ; samuel.bernard@unilim.fr

* Correspondence: iwamoto.yuji@nitech.ac.jp; Tel.: +81-52-735-5276

Results

Table S1. Chemical composition of 600 °C heat-treated Si-Co-O powder samples.

Sample Co/Si	Composition/ wt.%*			Co/Si Atomic Ratio	
	Si	Co	O	Cal.	Obs.
1/8	37.9	9.8	52.2	0.125	0.123
1/4	33.0	17.1	49.8	0.250	0.247
1/2	26.1	26.9	46.9	0.500	0.491

* Carbon content < 0.1 %.

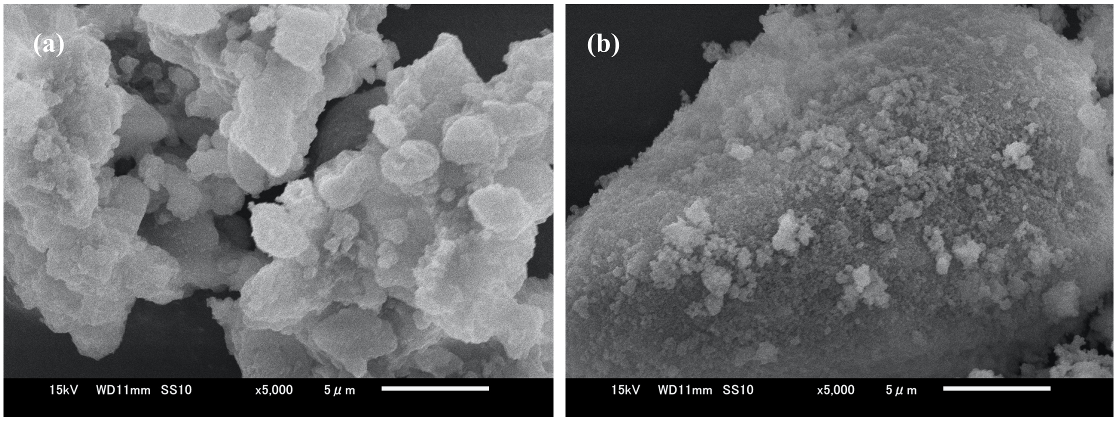

Figure S1. SEM images of the (**a**) CoSiOAlpow and (**b**) CoSiOAlpow2 samples.

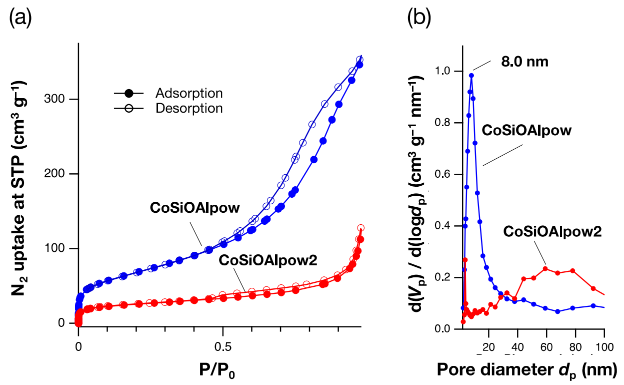

Figure S2. (**a**) N_2 adsorption-desorption isotherms at -196 °C for the CoSiOAlpow and CoSiOAlpow2 samples, and (**b**) the pore size distribution curves characterized by the BJH plot [1].

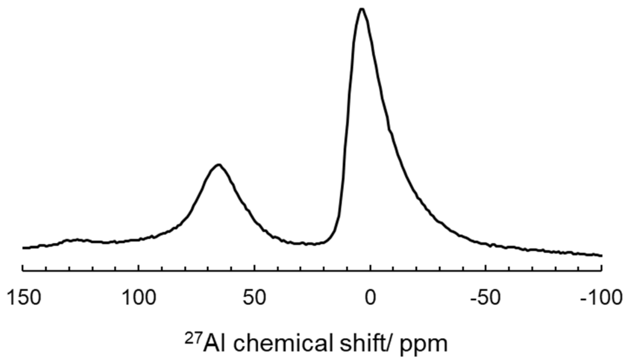

Figure S3. ^{27}Al solid–state MAS–NMR spectrum of the γ–Al_2O_3 powder sample showing two peaks at 66 and 1 ppm assigned to AlO_4 and AlO_6 units, respectively.

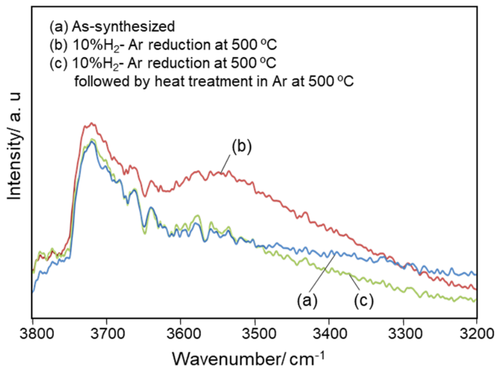

Figure S4. In situ DRIFT spectroscopic analysis for the Co-doped silica/γ–Al_2O_3 composite powder sample (CoSiOAlpow sample) with nominal composition of Al:Si:Co = 85:10:5. (**a**) As-synthesized, (**b**) after heat-treatment at 500 °C under 10% H_2–Ar flow for 8 h, and (**c**) after subsequent heat-treatment at 500 °C under Ar flow for 8 h.

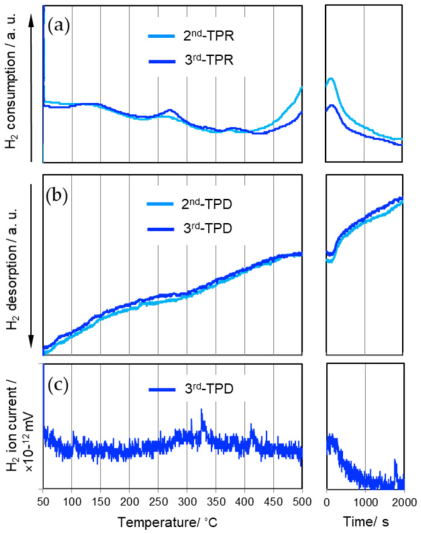

Figure S5. Results of cyclic TPR/TPD analysis for the Co-doped silica/γ–Al_2O_3 composite powder sample (CoSiOAlpow sample) with nominal composition of Al:Si:Co = 85:10:5. (**a**) 2nd and 3rd TPR profiles, (**b**) 2nd and 3rd TPD profiles, and (**c**) H_2 ion current detected during the 3rd TPD profile measurement.

References

1. Barrett, E.P.; Joyner, L.G.; Halenda, P.P. The Determination of Pore Volume and Area Distributions in Porous Substances. I. Computations from Nitrogen Isotherms, *J. Am. Chem. Soc.* **1951**, *73*, 373–380, doi.org/10.1021/ja01145a126.
